# Dataset on aerosol loading, size and statistics over Nzerekore

**DOI:** 10.1016/j.dib.2018.08.116

**Published:** 2018-08-30

**Authors:** M.E. Emetere, J.M. Emetere, O. Dauda

**Affiliations:** a Department of Physics, Covenant University Canaan land, P.M.B 1023, Ota, Nigeria; b Department of Mathematics, Federal University of Technology, Minna, Nigeria; c Department of Mechanical Engineering and Science, University of Johannesburg, APK, South Africa

**Keywords:** Aerosol loading, Aerosol, Nzerekore, Guinea, Model

## Abstract

Inadequate ground measuring equipment is becoming a global challenge. Most communities in West Africa rely only on satellite measurements. However, the volume of missing dataset in satellite measure over coastal communities is alarming. In this research, fifteen years primary (aerosol optical depth) dataset was obtained from the Multi-angle Imaging Spectro-Radiometer (MISR). The dataset presented in this research will serve as a research background for scientists working on the research site.

**Specifications table**

**Table:** 

Subject area	Air Pollution
More specific subject area	Aerosol loading and Retention,aerosol size and aerosol optical depth statistics
Type of data	Table and figure
How data was acquired	Multi-angle Imaging Spectro-Radiometer (MISR).
Data format	Raw and analyzed
Experimental factors	Data retrieval of Aerosol Optical Depth, data processing and statistics
Experimental features	Measurement at 550 nm
Data source location	Nzerekore-Guinea
Data accessibility	https://l0dup05.larc.nasa.gov/L3Web/download

**Value of the data**

• The data gives a good background for further study on atmospheric aerosol research.
• The data provides basis for modelling health hazard on inhalation.
• The data helps to quantify the extent of air pollution.
• The data provides a platform to validate data from emerging models.

## 1 Data

The volume of dataset loss on a yearly basis from satellite measurements over coastal regions of West Africa is alarming. Also, little or no presence of ground measuring equipments or exploration further contributes to the uncertainty of air pollution. Undoubteldy, the effect of air pollution is clearly seen by the increased rate of respiratory diseases and death [1]. Ref [2], [3], [4] have been able to estimate the aerosol loading trends and danger over neighbouring communities. The summarized primary data was obtained from Multi-angle Imaging Spectro-Radiometer (MISR) i.e. found in Table 1A, Table 1B, Table 1C for 550 nm wavelength [5]. The aersosl optical depth is found in Table 1A, Table 1B, Table 1C. MISR) i.e. found in Table 1A, Table 1B, Table 1C for 550 nm wavelength [5]. The aersosl optical depth is found in Table 1A, Table 1B, Table 1C. The empty spaces in Table 1A, Table 1B, Table 1C are the missing data perculiar to West Africa. Ref [6] revealed that the missing data is due to moisture, precipitation rate and cloud cover over West Africa.Aerosol loading over the area was obtained using the West African regional scale dispersion model [6] from the primary dataset (Table 2A, Table 2B, Table 2C). The Angstrom parameter is presented in Table 3A, Table 3B, Table 3C. The radius of particulateusing the back-envelope cateria is shown in Table 4A, Table 4B, Table 4C. The radius of the particulate that determine the level of danger of aerosols by inhalation is presented in Table 5A, Table 5B, Table 5C. The empty spaces in Table 2A, Table 2B, Table 2C, Table 3A, Table 3B, Table 3C, Table 4A, Table 4B, Table 4C, Table 5A, Table 5B, Table 5C is due to missing data that has been explained above The statistical analysis of the summarized promary dataset is shown in Table 6A, Table 6B, Table 6C.

**Table 1A:** Summarized Aerosol Optical Depth Dataset over Nzerekore for year 2000–2004.

**Month**	**2000**	**2001**	**2002**	**2003**	**2004**
**Jan**	1.036	0.441	0.394	0.496	0.379
**Feb**		0.450	0.391	0.407	0.793
**Mar**		0.348	0.488	0.461	0.864
**Apr**			0.291		0.471
**May**			0.211	0.203	
**Jun**					
**Jul**					
**Aug**					
**Sep**	0.222	1.079			
**Oct**	0.312	0.195			0.286
**Nov**				0.332	0.328
**Dec**		1.068	0.267	0.359	0.541

**Table 1B:** Summarized Aerosol Optical Depth Dataset over Nzerekore for year 2008–2009.

**Month**	**2005**	**2006**	**2007**	**2008**	**2009**
**Jan**	0.545	0.331	0.569	0.288	0.414
**Feb**	0.482	0.413	0.429	0.471	0.373
**Mar**	0.720	0.842	0.687	0.274	0.578
**Apr**	0.190				
**May**				0.323	0.322
**Jun**					
**Jul**					
**Aug**					
**Sep**					0.806
**Oct**				0.161	
**Nov**		0.144	0.397	0.181	0.232
**Dec**	0.471	0.409	0.421	0.398	0.247

**Table 1C:** Summarized Aerosol Optical Depth Dataset over Nzerekore for year 2010–2013.

**Month**	**2010**	**2011**	**2012**	**2013**
**Jan**	0.323	0.551	0.399	0.192
**Feb**	0.300	0.400	0.796	0.588
**Mar**	1.018	1.188	0.641	0.261
**Apr**	0.318	0.325	0.437	
**May**			0.257	
**Jun**				
**Jul**	0.095			
**Aug**				
**Sep**	0.348	0.225		
**Oct**	0.164	0.272	0.441	
**Nov**		0.473	0.275	0.253
**Dec**	0.630	0.508	0.307

**Table 2A:** Aerosol loading over Nzerekore between years 2000 and 2004.

**Month**	**2000**	**2001**	**2002**	**2003**	**2004**
**Jan**	0.558	0.864	0.880	0.844	0.884
**Feb**	0.944	0.861	0.881	0.876	0.702
**Mar**	0.944	0.894	0.847	0.857	0.661
**Apr**	0.944	0.944	0.909	0.944	0.853
**May**	0.944	0.944	0.925	0.927	0.944
**Jun**	0.944	0.944	0.944	0.944	0.944
**Jul**	0.944	0.944	0.944	0.944	0.944
**Aug**	0.944	0.944	0.944	0.944	0.944
**Sep**	0.923	0.531	0.944	0.944	0.944
**Oct**	0.904	0.928	0.944	0.944	0.910
**Nov**	0.944	0.944	0.944	0.898	0.899
**Dec**	0.944	0.538	0.914	0.891	0.826

**Table 2B:** Aerosol loading over Nzerekore between years 2005 and 2009.

**Month**	**2005**	**2006**	**2007**	**2008**	**2009**
**Jan**	0.824	0.898	0.813	0.909	0.873
**Feb**	0.849	0.874	0.868	0.853	0.887
**Mar**	0.741	0.674	0.758	0.913	0.810
**Apr**	0.929	0.944	0.944	0.944	0.944
**May**	0.944	0.944	0.944	0.901	0.901
**Jun**	0.944	0.944	0.944	0.944	0.944
**Jul**	0.944	0.944	0.944	0.944	0.944
**Aug**	0.944	0.944	0.944	0.944	0.944
**Sep**	0.944	0.944	0.944	0.944	0.694
**Oct**	0.944	0.944	0.944	0.933	0.944
**Nov**	0.944	0.935	0.879	0.930	0.922
**Dec**	0.853	0.875	0.871	0.879	0.919

**Table 2C:** Aerosol loading over Nzerekore between years 2010 and 2013.

**Month**	**2010**	**2011**	**2012**	**2013**
**Jan**	0.901	0.821	0.878	0.929
**Feb**	0.907	0.878	0.700	0.805
**Mar**	0.569	0.462	0.780	0.916
**Apr**	0.902	0.900	0.866	0.944
**May**	0.944	0.944	0.917	0.944
**Jun**	0.944	0.944	0.944	0.944
**Jul**	0.940	0.944	0.944	0.944
**Aug**	0.944	0.944	0.944	0.944
**Sep**	0.894	0.923	0.944	0.944
**Oct**	0.933	0.913	0.864	0.944
**Nov**	0.944	0.853	0.912	0.917
**Dec**	0.786	0.839	0.905	0.944

**Table 3A:** Angstrom parameter over Nzerekore between years 2000 and 2004.

**Month**	**2000**	**2001**	**2002**	**2003**	**2004**
**Jan**	-0.006	0.130	0.148	0.111	0.153
**Feb**		0.126	0.148	0.142	0.037
**Mar**		0.167	0.114	0.122	0.023
**Apr**			0.195		0.119
**May**			0.246	0.252	
**Jun**					
**Jul**					
**Aug**					
**Sep**	0.238	-0.012			
**Oct**	0.185	0.259			0.198
**Nov**				0.175	0.176
**Dec**		-0.010	0.209	0.162	0.097

**Table 3B:** Angstrom parameter over Nzerekore between years 2005 and 2009.

**Month**	**2005**	**2006**	**2007**	**2008**	**2009**
**Jan**	0.096	0.175	0.089	0.197	0.139
**Feb**	0.116	0.140	0.134	0.119	0.156
**Mar**	0.052	0.027	0.059	0.205	0.087
**Apr**	0.263				
**May**				0.179	0.180
**Jun**					
**Jul**					
**Aug**					
**Sep**					0.034
**Oct**				0.289	
**Nov**		0.307	0.146	0.270	0.231
**Dec**	0.119	0.142	0.137	0.146	0.222

**Table 3C:** Angstrom parameter over Nzerekore between years 2010 and 2013.

**Month**	**2010**	**2011**	**2012**	**2013**
**Jan**	0.179	0.094	0.145	0.261
**Feb**	0.191	0.145	0.036	0.084
**Mar**	-0.003	-0.027	0.070	0.212
**Apr**	0.182	0.178	0.131	
**May**			0.215	
**Jun**				
**Jul**	0.373			
**Aug**				
**Sep**	0.167	0.236		
**Oct**	0.286	0.206	0.130	
**Nov**		0.119	0.204	0.217
**Dec**	0.073	0.107	0.187

**Table 4A:** Radius of particulate-back of envelope calculation between years 2000 and 2004.

**Month**	**2000**	**2001**	**2002**	**2003**	**2004**
**Jan**	0.606	0.478	0.463	0.494	0.458
**Feb**		0.481	0.462	0.467	0.562
**Mar**		0.447	0.491	0.484	0.576
**Apr**			0.426		0.487
**May**			0.389	0.385	
**Jun**					
**Jul**					
**Aug**					
**Sep**	0.395	0.613			
**Oct**	0.434	0.381			0.424
**Nov**				0.441	0.440
**Dec**		0.611	0.416	0.451	0.506

**Table 4B:** Radius of particulate-back of envelope calculation between years 2005 and 2009.

**Month**	**2005**	**2006**	**2007**	**2008**	**2009**
**Jan**	0.507	0.441	0.513	0.424	0.470
**Feb**	0.490	0.469	0.474	0.487	0.456
**Mar**	0.548	0.572	0.541	0.418	0.515
**Apr**	0.378				
**May**				0.438	0.438
**Jun**					
**Jul**					
**Aug**					
**Sep**					0.565
**Oct**				0.361	
**Nov**		0.350	0.464	0.373	0.400
**Dec**	0.487	0.468	0.472	0.464	0.406

**Table 4C:** Radius of particulate-back of envelope calculation between years 2010 and 2013.

**Month**	**2010**	**2011**	**2012**	**2013**
**Jan**	0.438	0.508	0.465	0.379
**Feb**	0.429	0.465	0.563	0.518
**Mar**	0.603	0.629	0.530	0.413
**Apr**	0.436	0.439	0.476	
**May**			0.411	
**Jun**				
**Jul**	0.312			
**Aug**				
**Sep**	0.447	0.396		
**Oct**	0.363	0.418	0.478	
**Nov**		0.487	0.419	0.409
**Dec**	0.528	0.497	0.432

**Table 5A:** Radius of particulate-atmospheric aerosols between years 2000 and 2004.

**Month**	**2000**	**2001**	**2002**	**2003**	**2004**
**Jan**	8.480E-07	5.608E-07	5.354E-07	5.892E-07	5.276E-07
**Feb**		5.655E-07	5.342E-07	5.424E-07	7.340E-07
**Mar**		5.101E-07	5.852E-07	5.714E-07	7.677E-07
**Apr**			4.767E-07		5.764E-07
**May**			4.253E-07	4.197E-07	
**Jun**					
**Jul**					
**Aug**					
**Sep**	4.328E-07	8.680E-07			
**Oct**	4.890E-07	4.140E-07			4.737E-07
**Nov**				5.006E-07	4.986E-07
**Dec**		8.628E-07	4.619E-07	5.163E-07	6.118E-07

**Table 5B:** Radius of particulate-atmospheric aerosols between years 2005 and 2009.

**Month**	**2005**	**2006**	**2007**	**2008**	**2009**
**Jan**	7.361E-07	5.003E-07	6.261E-07	4.748E-07	5.466E-07
**Feb**	7.187E-07	5.456E-07	5.542E-07	5.763E-07	5.238E-07
**Mar**	7.777E-07	7.575E-07	6.838E-07	4.660E-07	6.303E-07
**Apr**	6.048E-07				
**May**				4.954E-07	4.948E-07
**Jun**					
**Jul**					
**Aug**					
**Sep**					7.404E-07
**Oct**				3.884E-07	
**Nov**		3.742E-07	5.372E-07	4.037E-07	4.395E-07
**Dec**	7.155E-07	5.436E-07	5.502E-07	5.378E-07	4.490E-07

**Table 5C:** Radius of particulate-atmospheric aerosols between years 2010 and 2013.

**Month**	**2010**	**2011**	**2012**	**2013**
**Jan**	4.959E-07	6.169E-07	5.383E-07	4.118E-07
**Feb**	4.820E-07	5.388E-07	7.356E-07	6.355E-07
**Mar**	8.396E-07	9.186E-07	6.614E-07	4.584E-07
**Apr**	4.925E-07	4.969E-07	5.584E-07	
**May**			4.554E-07	
**Jun**				
**Jul**	3.288E-07			
**Aug**				
**Sep**	5.101E-07	4.348E-07		
**Oct**	3.908E-07	4.651E-07	5.606E-07	
**Nov**		5.772E-07	4.670E-07	4.532E-07
**Dec**	6.562E-07	5.952E-07	4.865E-07

**Table 6A:** AOD statistics over Nzerekore between years 2000 and 2004.

**Statistics**	**2000**	**2001**	**2002**	**2003**	**2004**
Number of values	3.000	6.000	6.000	6.000	7.000
Number of missing values	9.000	6.000	6.000	6.000	5.000
Mean	0.523	0.597	0.340	0.376	0.523
First quartile	#N/A	0.348	0.267	0.332	0.341
Third quartile	#N/A	1.068	0.394	0.461	0.730
Standard error	0.258	0.155	0.042	0.043	0.085
95% confidence interval	1.109	0.399	0.107	0.110	0.209
99% confidence interval	2.558	0.626	0.168	0.172	0.317
Variance	0.199	0.145	0.010	0.011	0.051
Average deviation	0.342	0.318	0.084	0.078	0.179
Standard deviation	0.446	0.380	0.102	0.105	0.226
Coefficient of variation	0.853	0.637	0.299	0.278	0.432
Skew	1.654	0.705	0.235	-0.768	0.714
Kurtosis	#N/A	-1.763	-1.002	0.528	-1.147
Kolmogorov-Smirnov stat	0.349	0.317	0.192	0.168	0.184
Critical K-S stat, alpha=.10	0.636	0.468	0.468	0.468	0.436
Critical K-S stat, alpha=.05	0.708	0.519	0.519	0.519	0.483
Critical K-S stat, alpha=.01	0.829	0.617	0.617	0.617	0.576

**Table 6B:** AOD statistics over Nzerekore between years 2005 and 2009.

**Statistics**	**2005**	**2006**	**2007**	**2008**	**2009**
Number of values	5.000	5.000	5.000	7.000	7.000
Number of missing values	7.000	7.000	7.000	5.000	5.000
Minimum	0.481	0.428	0.501	0.299	0.424
First quartile	0.401	0.284	0.415	0.204	0.265
Third quartile	0.588	0.520	0.599	0.379	0.537
Standard error	0.085	0.115	0.056	0.042	0.077
95% confidence interval	0.237	0.318	0.154	0.102	0.189
99% confidence interval	0.393	0.528	0.256	0.155	0.287
Variance	0.036	0.066	0.015	0.012	0.042
Average deviation	0.121	0.166	0.102	0.084	0.153
Standard deviation	0.191	0.256	0.124	0.111	0.205
Coefficient of variation	0.397	0.599	0.248	0.370	0.482
Skew	-0.656	1.176	1.051	0.294	1.232
Kurtosis	1.828	2.491	-0.564	-0.678	1.063
Kolmogorov-Smirnov stat	0.278	0.323	0.319	0.143	0.234
Critical K-S stat, alpha=.10	0.509	0.509	0.509	0.436	0.436
Critical K-S stat, alpha=.05	0.563	0.563	0.563	0.483	0.483
Critical K-S stat, alpha=.01	0.669	0.669	0.669	0.576	0.576

**Table 6C:** AOD statistics over Nzerekore between years 2010 and 2013.

**Statistics**	**2010**	**2011**	**2012**	**2013**
Number of values	8.000	8.000	8.000	4.000
Number of missing values	4.000	4.000	4.000	8.000
Mean	0.399	0.493	0.444	0.324
First quartile	0.232	0.299	0.291	0.223
Third quartile	0.489	0.529	0.541	0.425
Standard error	0.104	0.107	0.066	0.089
95% confidence interval	0.247	0.254	0.157	0.285
99% confidence interval	0.365	0.375	0.232	0.523
Variance	0.087	0.092	0.035	0.032
Average deviation	0.212	0.192	0.137	0.132
Standard deviation	0.295	0.303	0.188	0.179
Coefficient of variation	0.738	0.616	0.423	0.553
Skew	1.525	2.053	1.068	1.816
Kurtosis	2.393	4.897	0.396	3.487
Kolmogorov-Smirnov stat	0.319	0.299	0.257	0.386
Critical K-S stat, alpha=.10	0.410	0.410	0.410	0.565
Critical K-S stat, alpha=.05	0.454	0.454	0.454	0.624
Critical K-S stat, alpha=.01	0.542	0.542	0.542	0.734

## 2 Experimental design, materials and methods

**Fig. 1:**
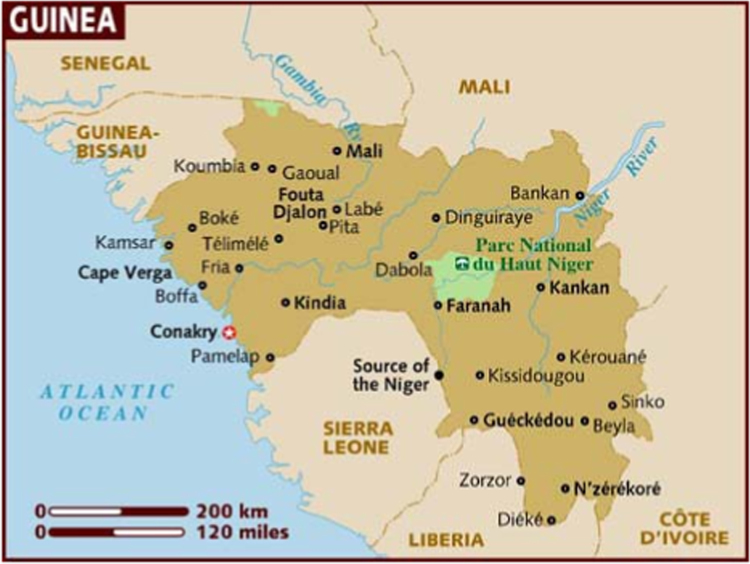
Geographical map of Nzerekore.

Nzerekore is located in Guinea on longitude and latitude of 8.8253 °W and 7.7478 °N (Fig. 1).

The West African regional scale dispersion model (WASDM) for calculating aerosol loading over a region [6]:

(1) $$\psi \left(\lambda \right)={a_{1}}^{2}\cos\left(\frac{n_{1}\pi \tau (\lambda )}{2}x\right)\cos\left(\frac{n_{1}\pi \tau (\lambda )}{2}y\right)+\ldots \ldots {a_{n}}^{2}\cos\left(\frac{n_{n}\pi \tau (\lambda )}{2}x\right)\cos\left(\frac{n_{n}\pi \tau (\lambda )}{2}y\right)$$

a is atmospheric constant gotten from the fifteen years aerosol optical depth (AOD) dataset from MISR, n is the tunning constant, τ(λ) is the AOD of the area and ψ(λ) is the aerosol loading. The data processing was done using the excel. The validation of the summarized dataset was done using mathematical models and statistical softwares. The analysis of Eq. (1) was done using the C++ codes.
